# Rapid doubling of Alzheimer’s amyloid-β40 and 42 levels in brains of mice exposed to a nickel nanoparticle model of air pollution

**DOI:** 10.12688/f1000research.1-70.v1

**Published:** 2012-12-21

**Authors:** Soong Ho Kim, Elysse M Knight, Eric L Saunders, Azita K Cuevas, Marusia Popovech, Lung-Chi Chen, Sam Gandy

**Affiliations:** 1 Department of Neurology, Icahn School of Medicine at Mount Sinai, New York, NY 10029, USA; 2 Department of Environmental Sciences, New York University, Tuxedo Park, New York, NY 10987, USA; 3 Department of Psychiatry and Alzheimer’s Disease Research Center, Icahn School of Medicine at Mount Sinai, New York, NY 10029, USA; 4 James J. Peters Veterans Affairs Medical Center, Bronx, New York, NY 10468, USA

## Abstract

**Background:** Over 20 genetic risk factors have been confirmed to associate with elevated risk for Alzheimer’s disease (AD), but the identification of environmental and/or acquired risk factors has been more elusive. At present, recognized acquired risks for AD include traumatic brain injury, hypercholesterolemia, obesity, hypertension, and type 2 diabetes.

**Methods:** Based on reports associating various inhalants with AD pathology, we investigated the possibility that air pollution might contribute to AD risk by exposing wild-type mice to a standard air pollution modeling system employing nickel nanoparticle-enriched atmosphere for 3 hr.

**Results:** Mice exposed to air pollution showed 72-129% increases in brain levels of both amyloid-β peptides Aβ40 and Aβ42, as well as Aβ42/40 (p <0.01).

**Conclusions:** These effects on elevation of brain Aβ exceed those associated with trisomy 21, a known risk for early onset AD pathology, raising the possibility that clinical importance might be attached. Further work is required to establish the molecular and physiological basis for these phenomena. The rapid, dramatic effect, if verified, would suggest that inhalant exposures should be evaluated for their possible roles in contributing to the environmental risk for common forms of AD.

## Introduction

One common neurodegenerative disease, Parkinson’s disease, has been linked to exposure to MPTP (1-methyl-4-phenyl-1,2,3,6-tetrahydropyridine) and to inhaled manganese ^1, 2^. Similarly, inhaled aluminum dust has been associated with neurotoxic effects and pre-clinical cognitive impairment ^3^. Certain inhalation anesthetics have also been implicated in elevating AD risk, possibly by exacerbating the neurotoxic oligomerization of the amyloid-β (Aβ) peptide ^4^. The early involvement of the olfactory cortex in AD has caused longtime speculation that some inhaled agent might play a role in AD risk ^5^.

Recently, AD pathology was identified in young people living in areas with high levels of air pollution ^6, 7^. Furthermore, impaired cognition has been recently attributed to air pollution exposure in some populations ^8^. These converging lines of evidence led us to analyze brain levels of Aβ40 and Aβ42 in mice exposed to an inhaled particulate matter (nickel nanoparticle; Ni NP) model of air pollution.

## Methods

All procedures involving animals were conducted in compliance with guidelines for ethical animal research and approved by the New York University School of Medicine Animal Care and Use Committee. Two-month-old male and female FVBN mice (Taconic Farm, Hudson, NY) were randomly assigned to Ni NP inhalation (count median diameter 54 nm, at 1 mg/m ^3^, which is the current Occupational Safety and Health Administration’s Permissible Exposure Limit for nickel hydroxide [ http://www.osha.gov/pls/oshaweb/owadisp.show_document?p_table=standards&p_id=9992]) ( *n* = 16 per group) or control filtered air ( *n* = 5 per group) for 3 hours in a nose-only exposure chamber. This protocol has been established as a model for air pollution toxicity in pulmonary disease ^9^, atherosclerosis ^10^, and insulin resistance ^11^. Twenty-four hours post exposure, mice were given pentobarbital, bled out via the vena cava, and then their brains were harvested, snap frozen and stored at -80ºC until assay. For measurement of endogenous mouse brain Aβ40 and Aβ42, we employed the Schmidt method ^12^ and human/rat Aβ 1–40/1–42 ELISA kits (Wako, Richmond, VA). Statistical analysis was performed via Mann-Whitney test. #8 Ni NP is excluded from the analysis due to being more than 2 SD's away from mean or closest value.

## Results

**Figure 1.**
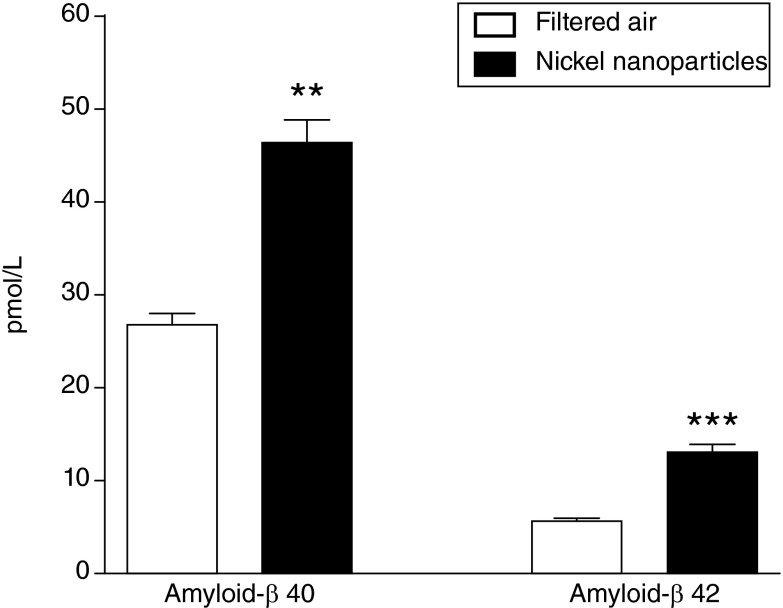
Exposure to air pollution increases amyloid-β (Aβ) levels in the mouse brain. Elevated endogenous mouse brain Aβ40 and Aβ42 in mice exposed to nickel nanoparticles (count median diameter 54 nm, at 1 mg/m ^3^) ( *n* = 16 per group) versus filtered air ( *n* = 5 per group) for 3 hours in a nose-only exposure chamber. Data presented as mean + SEM. ** *P* < 0.01, *** *P* < 0.001 (Mann-Whitney test).

Both endogenous Aβ40 and Aβ42 were elevated in the brains of mice following Ni NP exposure ( Figure 1). Aβ40 was increased by 1.72-fold ( *P* = 0.0011, Mann-Whitney test), and Aβ42 was increased by 2.29-fold ( *P* = 0.0005, Mann-Whitney test). Aβ42/40 ratio was also increased in the Ni NP-exposed group compared to the filtered air control group (0.27 ± 0.01 and 0.21 ± 0.007, respectively; P = 0.0093, Mann-Whitney test). Both male and female mice responded similarly to Ni NP exposure (male *vs*. female for Aβ40 and Aβ42 levels; *P* > 0.1, Mann-Whitney test).

Raw data table for endogenous mouse brain Aβ40 and Aβ42 levels in mice exposed to nickel nanoparticles versus filtered air. Raw data of endogenous mouse brain Aβ40 and Aβ42 levels (pmol/L) in mice exposed to nickel nanoparticles (count median diameter 54 nm, at 1 mg/m^3^) (n = 16 per group) versus filtered air (n = 5 per group) for 3 hours in a nose-only exposure chamber. *Mouse 8 was excluded from the data analysis as these results were 2 SD's away from mean or closest value.

## Discussion

These data add credence to the proposal ^4^ that one or more inhaled neurotoxin(s) might increase the risk for AD by elevating levels of brain Aβ. We have not identified whether this accumulation occurs at the level(s) of transcription, translation, or post-translational processing. It is tempting to speculate that the well-known links between inhaled toxins and brain inflammation, and other links between brain inflammation and AD established by Griffin and colleagues ^13^ may underlie these phenomena.

The changes that we observed were dramatic, rapid, and unexpected. Human Aβ is more aggregatable than murine Aβ, making it conceivable that the effect on Aβ levels in human brain could be even greater. While elucidating the genesis and molecular underpinnings will be an important next step, an even more important step will be a rigorous application of environmental toxicology and epidemiology to determine whether the elevated brain Aβ caused in mice by this air pollution model corresponds to any situation of authentic human inhalation exposure that is linked to an increased risk for AD.
