# Supplementary material for: Rapid doubling of Alzheimer’s amyloid-β40 and 42 levels in brains of mice exposed to a nickel nanoparticle model of air pollution
Source: F1000Res. 2012 Dec 21;1:70. [Version 1] doi: 10.12688/f1000research.1-70.v1 (PMC3782349; doi:10.12688/f1000research.1-70.v1)
Supplement: Peer review history

# Peer review: Referee response for version 1

**Published:** 2013-01-25

**Authors:** Ottavio Arancio

**Affiliations:** 1 Columbia University Medical Center, New York, NY, USA

This is an intriguing observation that might potentially have important relevance to the etiopathogenesis of Alzheimer’s Disease.

I have read this submission. I believe that I have an appropriate level of expertise to confirm that it is of an acceptable scientific standard.

# Peer review: Referee response for version 1

**Published:** 2013-01-23

**Authors:** George Perry

**Affiliations:** 1 University of Texas at San Antonio, San Antonio, TX, USA

Linkage of environmental air toxicity from nanoparticles leading to Alzheimer-like changes in the brain of mice opens a new avenue to understanding the development of brain diseases. These findings are consistent with prior work showing particulate air pollution can affect the brains of human as well as dogs living in polluted cities.

I have read this submission. I believe that I have an appropriate level of expertise to confirm that it is of an acceptable scientific standard.

# Peer review: Referee response for version 1

**Published:** 2013-01-21

**Authors:** W Sue Griffin

**Affiliations:** 1 Department of Geriatrics, University of Arkansas for Medical Sciences, Little Rock, AR, USA

Neurodegenerative consequences are a looming possibility with the current increased burden of air-borne pollutants. Many studies have suggested an association between anesthesia administered to older adults and cognitive decline and development or progression of Alzheimer’s disease (AD). A number of studies have already established links between air-borne pollutants and risk for development of systemic disorders several of which, like AD are inflammatory in nature. Gandy and his colleagues have taken a purposeful step toward more directly connecting air-pollution to increased risk for development of AD in a study of wild type mice exposed to a toxin at the OSHA permissible level for an 8h human exposure. The results are very convincing, showing that a one-time 3h exposure to nickel hydroxide nanoparticles at the “permissible” level doubled brain levels of Aβ40 and Aβ42 within 24h—increases that are similar to levels reported in younger adults with Down’s syndrome. Interestingly, as noted by the authors, and in accord with systemic inflammatory consequences reported following exposure to air-borne pollutants, neuroinflammatory changes that drive Alzheimer neuropathological change may also be elicited by such exposure. This may be particularly important as we necessarily inhale air that appears to be “clean,” but which contains permissible levels of agents that may have adverse effects on the brain, especially in persons with genetic risk factors and or co-morbid conditions that already predispose them for development of AD.

I have read this submission. I believe that I have an appropriate level of expertise to confirm that it is of an acceptable scientific standard.
